# Virulent *Leptospira interrogans* Induce Cytotoxic Effects in Human Platelets *in vitro* Through Direct Interactions

**DOI:** 10.3389/fmicb.2020.572972

**Published:** 2020-09-24

**Authors:** Monica Larucci Vieira, Ana Lucia T. O. Nascimento

**Affiliations:** ^1^Department of Microbiology, Institute of Biological Sciences, Federal University of Minas Gerais (UFMG), Belo Horizonte, Brazil; ^2^Laboratório de Desenvolvimento de Vacinas, Butantan Institute, São Paulo, Brazil

**Keywords:** leptospirosis, *Leptospira*, blood platelets, platelet aggregation, hemostasis, host-pathogen interactions, infectious diseases, spirochetes

## Abstract

Leptospirosis is a prevalent zoonotic disease, caused by bacteria of the genus *Leptospira*. Leptospirosis frequently leads to hemostatic disturbances, and the severe cases are marked by hemorrhages and low platelet number in circulation, which is associated with the patients’ poor outcomes. Nevertheless, *Leptospira*-platelet interactions remain poorly explored. In this study, we performed a series of *in vitro* experiments evaluating whether leptospires induce human platelet aggregation, activation, and morphological changes. Platelets were incubated with virulent *L. interrogans* and the platelet outcomes were assessed by aggregometry, flow cytometry, and scanning and transmission electron microscopy. Our results show that leptospires alone do not induce platelet aggregation and activation, and induce platelet cytotoxic effects instead, by clearly inducing platelet disruption and detachment. We show for the first time that virulent leptospires do interact directly with platelets, an event that could trigger pathophysiological effects during the infection. This study might serve as a basis for the development of novel treatments for the disease.

## Introduction

Leptospirosis is one of the most globally important zoonosis, lately considered a leading re-emerging infectious disease both in developing and developed countries (Dupouey et al., 2014; Pijnacker et al., 2016). Pathogenic species of the genus *Leptospira* spp. cause the disease. It is believed that more than 1 million cases of symptomatic human leptospirosis cases occur yearly, reaching up to 60,000 deaths (Costa et al., 2015). The incidence of human leptospirosis is high in low and middle-income tropical countries, mostly related to flooding events and the lack of adequate sanitation. In high-income countries located in temperate regions, the occurrence of the disease is low and mostly related to occupational or water-related recreational activities (Hartskeerl et al., 2011). Marked increasing leptospirosis incidence rates and multiple outbreaks have been recently reported in all continents (Hartskeerl et al., 2011; Dupouey et al., 2014; Pijnacker et al., 2016). The disease also affects livestock, representing a significant economic burden.

Human infection usually occurs through contact with contaminated water or soil, or contact with infected animals’ fluids. In the initial phase of the disease, leptospiremia, bacteria reach the bloodstream, where they persist and multiply, then migrate into the surrounding tissue. When the host develops an immunological antibody response against the pathogen, leptospires are cleared from the blood, characterizing the immune phase or leptospiruria (Levett, 2001).

Human leptospirosis clinical manifestation ranges from asymptomatic to fatal forms. The infection usually presents initially as a non-specific, sudden onset illness with fever, chills, myalgia, abdominal pain, and headache. Leptospirosis can progress to a severe condition known as Weil’s syndrome, corresponding to 5–15% of reported cases, with a mortality rate of around 15% (Levett, 2001; Bharti et al., 2003). The complications of severe leptospirosis include jaundice, hypotension, acute lung injury, bleeding, and multi-organ failure. The leptospirosis-associated pulmonary hemorrhagic syndrome (LPHS), has been lately on the focus of attention. LPHS has been increasing worldwide, and the lethality can reach 70% (Toyokawa et al., 2011). Despite the importance of the disease, the mechanisms of leptospirosis pathogenesis at the molecular and cellular levels are not fully comprehended (Picardeau, 2017).

Although it is still unclear why leptospirosis patients can experience a range of different clinical symptoms, an increasing body of evidence indicates that compromised hemostasis is involved in the manifestation of the pathophysiology with an effect on the outcome of the disease (Vieira et al., 2020). Thrombocytopenia, the low platelet number in circulation, is a consistent finding in patients with severe leptospirosis and is associated with a worse prognosis and occurrence of hemorrhages (Davenport et al., 1989; Nicodemo et al., 1990; Wagenaar et al., 2007; Daher et al., 2014). A number of hypothesis have been raised to explain the decrease in blood platelet numbers during the disease, including over-activation and aggregation, increased platelet clearance, autoimmune-mediated platelet destruction, and action of unknown leptospiral toxins (Vieira et al., 2020). However, the underlying mechanisms of leptospirosis thrombocytopenia remain to be determined.

In the present study, we investigated the *Leptospira*-platelet interactions *in vitro*, analyzing the resulting platelet aggregation activity, activation status and morphological characteristics. Our results suggest a direct interaction of leptospires and platelets, and give an insight of the consequences of these interactions for the platelet function, integrity, and leptospirosis symptomatology.

## Materials and Methods

### Ethics Statement

All blood donors gave written informed consent. The study complied with the Declaration of Helsinki and was approved by the Ethics Committee on Research of Instituto de Assistência Médica ao Servidor Público Estadual (IAMSPE), São Paulo, Brazil – protocol 2973410.

### *Leptospira* Strain and Culture Conditions

Virulent *Leptospira interrogans* serovar Copenhageni strain L1-130, were kindly provided by Dr. Marcos Heinemann (University of São Paulo, Brazil). Leptospires were cultured at 28°C in Elinghausen-McCullough-Johnson-Harris (EMJH) medium (BD, Difco) supplemented with 10% *Leptospira* enrichment EMJH medium (BD, Difco), 0.3 g/L peptone (BD, Difco), and 0.2 g/L meat extract (Sigma-Aldrich). The bacteria were used up to the third passage for the maintenance of virulence.

### Platelet Rich Plasma Preparation

Peripheral human blood was collected by venipuncture onto sodium citrate vacuum tubes (BD). Platelet-rich plasma (PRP) was prepared by centrifugation of citrated blood at 140 × *g* for 15 min at room temperature (RT). The centrifugation was realized without brake to minimize platelet activation. The prepared PRP was immediately used.

### Washed Platelets

HEP buffer (140 mM NaCl, 2.7 mM KCl, 3.8 mM HEPES, 5 mM EGTA, pH 7.4) was added at 1:1 ratio (v/v) to PRP. Prostaglandin E1 (Sigma-Aldrich) was added to prevent platelet activation (2 μM final concentration). After a centrifugation step to pellet contaminating red and blood cells (100 × *g*; 15 min), the supernatant was collected and centrifuged to collect the platelets (800 × *g*; 10 min). The sediment was rinsed twice (10 mM sodium citrate, 150 mM NaCl, 1 mM EDTA, 1% (w/v) dextrose, pH 7.4) and suspended in HEPES Tyrode’s buffer (134 mM NaCl, 12 mM NaHCO_3_, 2.9 mM KCl, 0.34 mM Na_2_HPO_4_, 1 mM MgCl_2_, 10 mM HEPES, pH 7.4) containing 5 mM glucose, 3 mg/mL bovine serum albumin (BSA) and 1.8 mM CaCl_2_.

### Platelet Aggregation

Platelet-rich plasma was adjusted to approximately 1 × 10^8^ platelets/mL. PRP (400 μL) was displaced onto glass cuvettes. As positive control, platelet aggregation was initiated by adding final concentrations of 10 μM ADP (Chrono-Log). Platelet aggregation without adding the agonist was also recorded (negative control). *L. interrogans* (1 × 10^6^, 2 × 10^6^, 1 × 10^8^ or 1 × 10^9^ cells/mL) were added to stimulate platelets. Platelet aggregation was analyzed in a light transmission aggregometer (EasyAgreg, Qualiterm), up to 2 h at 37°C. The experiments were performed with four different blood donors, in triplicates. One-way ANOVA statistical analysis was performed to compare the *Leptospira*-treated samples with the positive-control (ADP-treated samples) (GraphPad Prism 8).

Alternatively, to access platelet reactivity, PRP was pre-incubated with the addition of leptospires (1 × 10^9^ cell/mL) or PBS (negative control) for 30 min. Afterward, 10 μM ADP were added and platelet aggregation was recorded up to 30 min, as described before. Unpaired two-tailed *t-*test was performed to compare the two data sets (GraphPad Prism 8).

### Flow Cytometry

After blood collection and PRP separation, samples were immediately processed for flow cytometry (FC). PRP (20 μL of 1 × 10^8^ cells/mL solution) was mixed with the antibodies cocktail (20 μL in PBS, phosphate-buffered saline) and the test stimulus (40 μL in PBS). As stimuli, we used buffer only (negative control), 10 μM ADP (positive control for platelet activation) or *L. interrogans* Copenhageni (2 × 10^6^ cells/mL). The reactions were incubated for 5, 10 or 30 min at RT, protected from light, then stopped and fixed by the addition of 500 μL 0.2% formaldehyde. The following conjugated antibodies dilutions were used: 5 μL CD41a-APC, 3 μL CD62P-BV421, 5 μL PAC-1-FITC, 5 μL CD63-FITC – all from BD Bioscience. Flow cytometric acquisition was performed with a FACS Canto II after optimization of settings using the cytometer setup and tracking beads. Instrument and reagents were from BD Bioscience. Data were analyzed using FlowJo version 7.6.5. The experiments were performed with four different blood donors, at least in duplicates. Data from 20,000 events in the CD41a-positive gate were collected for each sample.

### Scanning Electron Microscopy (SEM) of Pre-adhered Platelets

Platelet-rich plasma or washed platelet (100 μL of 1 × 10^8^ cells/mL solution) were disposed onto round glass coverslips treated with poly-L-lysine and allowed to adhere for 30 min. The coverslips were washed with buffer, following incubation for 30 min at RT with (i) buffer, (ii) 10 μM ADP, or *L. interrogans* Copenhageni (1 × 10^7^/mL) in 200 μL. After a washing step, the samples were fixed with Karnowsky buffer (glutaraldehyde 5% and paraformaldehyde 4% in 0.1 M sodium cacodilate buffer pH 7.2) for 3 h, then washed again with 0.1 M sodium cacodilate buffer pH 7.2. The coverslips were post-fixed in OsO_4_ 1% for 2 h. The fixed material was dehydrated in an ethanol gradient (70, 80, 90, and 100°GL), and critical point dried in CO_2_. Finally, the slides were taped onto stubs, layered with gold and observed under a FEI Quanta 200 scanning electron microscope. Two independent experiments were performed in duplicates, representing two different blood donors.

### SEM of Platelets Treated in Solution

Washed platelets (100 μL of 1 × 10^8^ cells/mL solution) were mixed with (i) buffer, (ii) 10 μM ADP, or *L. interrogans* Copenhageni (1 × 10^7^/mL) in 50 μL, and incubated for 30 min at RT. The cells and bacteria were then pelleted (1,000 × *g*, 10 min) and washed, following fixation in 200 μL Karnowsky for 3 h. The fixed samples were allowed to adhere onto coverslips treated with poly-L-lysine for 30 min, washed with 0.1 M sodium cacodilate buffer pH 7.2, and prepared for SEM as described above. Two independent experiments were performed in duplicates, with two different blood donors.

### SEM Image Platelet Cell Count and Histogram Analysis

The resting and dendritic cells visible as clear isolated entities (representing mostly the platelets in the over layer) in the SEM images were counted with the aid of the software ImageJ (version 1.53a). Two independent replicates were counted for each sample. ImageJ was also used for the evaluation of the gray-scale histograms, to indirectly quantify the cell detachment with consequent exposure of the glass surface and shift of the histogram profile to a darker hue.

### Transmission Electron Microscopy (TEM) of Platelets Treated in Solution

The washed platelets were treated with PBS, ADP, or leptospires as described above. The samples were fixed in suspension by adding 2.5% glutaraldehyde (final concentration) for 30 min, then pelleted (1,000 × *g*, 15 min). The precipitates were washed, then post-fixed in 1% osmium tetroxide, dehydrated, and embedded in epoxy resin. Ultrathin sections were cut in a Leica UC7 ultramicrotome, contrasted in 2% uranyl acetate and lead citrate. Grids were viewed with a LEO 906 E (Zeiss) transmission electron microscope, operating at maximum 120 Kv. The images were captured with the software ITEM.

### Determination of Mitochondrial Transmembrane Potential (Δψm) Depolarization

For measuring Δψm depolarization, platelets were incubated with DiOC6(3), a cell-penetrating green-fluorescing dye, and analyzed by flow cytometry. Depolarization results in a reduced DiOC6(3) accumulation in mitochondria when Δψm is dissipated. PRP aliquots were treated with, PBS (negative-control), 10 μM ADP, 5 U/mL thrombin (Sigma-Aldrich) or leptospires, as described before, with the addition of 100 nM DiOC6(3) (Sigma-Aldrich). The samples were diluted to 500 μL with PBS and flow cytometric acquisition was performed. Δψm depolarization was quantified as a decrease of the mean channel fluorescence (MCF) of platelet-bound DiOC6(3) (CD41a-positive gate). Two independent experiments were performed, in triplicates. One-way ANOVA statistical analysis was performed (GraphPad Prism 8).

## Results

### *L. interrogans* Is Unable to Induce Human Platelet Aggregation *in vitro*

To test whether pathogenic leptospires are able to induce human platelets to form aggregates *in vitro*, various *L. interrogans* serovar Copenhageni L1-130 MOI were mixed and incubated for different times with PRP. The formation of platelet aggregates was monitored in a light transmission aggregometer for up to 2 h (Table 1). No platelet aggregation was seen in the control with PBS while a typical aggregation response was observed after platelet treatment with ADP, a known agonist, indicating the viability of the cells. In the samples treated with the bacteria, no platelet aggregation was observed at any bacteria-to-platelet ratio after 2 h of incubation. Importantly, the tests include physiologically relevant bacteria numbers during leptospiremia (1 × 10^6^ or 2 × 10^6^/mL leptospires). Our data suggest that leptospires alone cannot induce human platelets to form aggregates *in vitro* in the assayed conditions.

**TABLE 1:** Platelet aggregation in response to different stimuli.

PBS	ADP	*Leptospira* (1 × 10^6^)	*Leptospira* (2 × 10^6^)	*Leptospira* (1 × 10^8^)	*Leptospira* (1 × 10^9^)
4.25 ± 1.26	87.25 ± 5.90	7.25 ± 3.77*	8.75 ± 2.97*	9.00 ± 4.08*	6.75 ± 2.50*

Platelets in PRP (approximately 1 × 10^8^ cells/mL) were mixed with PBS (negative control), ADP (positive control) or L. interrogans serovar Copenhageni in different densities (1 × 10^6^, 2 × 10^6^, 1 × 10^8^ or 1 × 10^9^ cells/mL) and the platelet aggregation was monitored in a light aggregometer for up to 2 h. The results represent the mean ± standard deviation of four independent experiments performed in duplicates, with different healthy blood donors. One-way ANOVA analysis was performed to compare the Leptospira-treated samples with the positive control sample (ADP). *P > 0.0001.

### Platelets Are Not Canonically Activated by *L. interrogans* Copenhageni *in vitro*

The next set of experiments were designed to further analyze the activation status of platelets after incubation with leptospires. The experiments were performed with PRP as platelet source to better represent physiological conditions during an infection.

**FIGURE 1:**
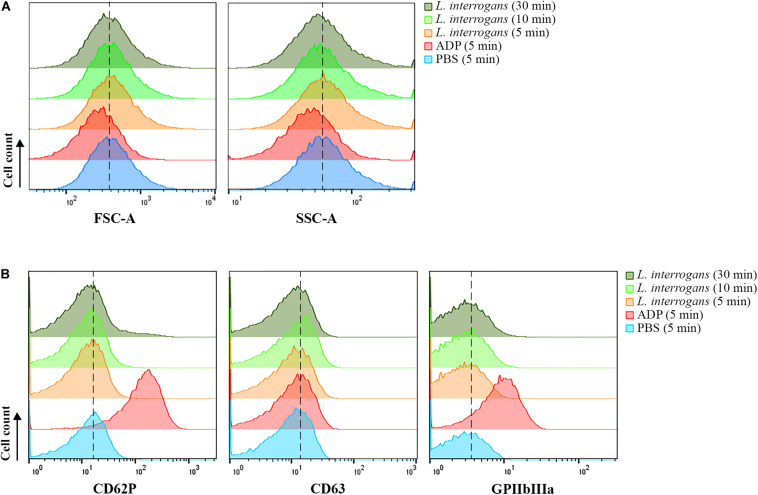
Flow cytometry analysis of platelets in PRP submitted to different stimuli. Platelets in PRP were incubated with the addition of PBS (negative control) or ADP (positive control) for 5 min, or virulent leptospires (2 × 10^6^/mL) for 5, 10 or 30 min. Platelets were marked with CD41a-APC and the results represent only the platelet gate. **(A)** SSC and FSC profiles of the CD41a-positive population. **(B)** CD62P (P-selectin), CD63, or activated GPIIbIIIa detection on the membrane of the CD41a-positive gate, respectively. The data are representative of four independent experiments, with four different healthy blood donors. For each sample, 20,000 events were collected. The dashed line was included to aid in the interpretation of the histogram displacement.

When activated, platelets undergo a shape change to a dendritic-like form, with cell membrane projections, following the degranulation and exposure of activation markers on the membrane, as well as conformational changes of receptors such as CD62P, CD63, and GPIIbIIIa. Human platelets were incubated with virulent leptospires, and the light scatter characteristics of the cell population was analyzed by FC as an indication of changes in the platelet morphology and granular content. We observed no significant changes in the SSC or FSC profiles of the CD41a-positive gate (platelets) after incubation with *L. interrogans* serovar Copenhageni for different times (5, 10, or 30 min), when compared to the negative control samples (buffer only) (Figure 1A). As expected, platelets treated with ADP as positive control for cell activation resulted in a change of the SSC and FSC profiles as soon as after 5 min incubation, compatible with a morphological change to an activated state and secretion of cytoplasmic granules (Figure 1A).

The analysis of platelet surface activation markers by FC revealed the absence of CD62P (P-selectin) and CD63 membrane exposure in the platelets (CD41a-positive gate) treated with *L. interrogans* or with buffer only (negative control) (Figure 1B). The activated state of the integrin GPIIbIIIa was also undetected both in the *Leptospira*-treated platelets and in the negative control (Figure 1B). As with the platelet aggregation assays, different bacteria densities and different incubation times were used, with similar results. The positive control samples, activated with ADP, showed increased exposure of P-selectin, CD63, and high GPIIbIIIa activation, as expected (Figure 1B). The low degree of CD63 secretion of ADP-treated platelets makes sense as CD63 becomes only exposed on strongly activated platelets, and ADP is a weak platelet agonist. Collectively, our data suggest that platelets are not canonically activated by *L. interrogans*.

### *L. interrogans* Induces Human Platelet De-Adherence and Cell Damage

**FIGURE 2:**
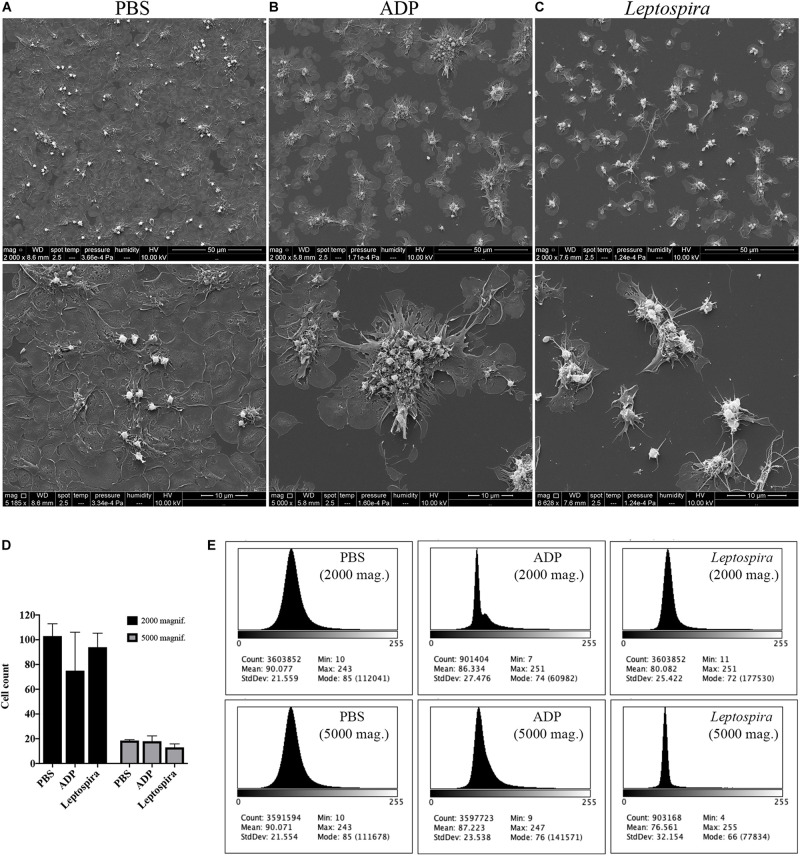
SEM showing the effect of leptospires on pre-adhered platelets. Platelets from PRP were allowed to adhere to coverslip glass surfaces, washed, and treated with PBS **(A)**, ADP (**B**; positive control), or leptospires **(C)** for 30 min. After washing and fixation, samples were prepared for scanning electron microscopy. Upper panel: 2,000 magnification. Lower panel: 5,000 magnification. The figure shows representative panels of the samples. Two independent experiments were performed, with similar results. **(D)** Cell count of resting or dendritic platelets observed as individual cells in SEM images. The bars represent the mean ± standard deviation of two independent experiments. **(E)** Gray-scale histograms of the representative images, as analyzed by the software ImageJ.

Human platelets in PRP were allowed to interact with glass coverslips previously treated with poly-L-lysine. This procedure results in spread and adhered cells. The pre-adhered platelets were then treated with *L. interrogans* suspensions, ADP (positive control for platelet activation and aggregation), or buffer alone (negative control). The cell morphology and cell-bacteria interactions were analyzed by SEM. Buffer-treated platelets maintained prevalent totally-spread or dendritic-spread morphology, covering all the glass surface (Figure 2A). ADP addition resulted in platelet shape change to spread-dendritic or dendritic-like state and formation of large platelet aggregates (Figure 2B). When treated with *L. interrogans* (Figure 2C), no totally spread cells were observed. Platelet retraction, cell fragmentation, and formation of small clusters or microaggregates (composed of platelets and bacteria) were also consistently observed. Direct platelet-bacteria interactions seem to occur.

As the enumeration of spread platelets is hindered by the impossibility to determine the limit of individual cells, especially in the confluent PBS samples, we counted (in independent duplicates) the resting and dendritic platelets visible as clear isolated cells, representing mostly the platelets in the over layer. As a measure of platelet detachment and consequent exposure of the glass surface with a shift in the gray-scale color, we analyzed the histograms of the images. The platelet count indicates that the number of resting and dendritic cells was similar between the different samples (Figure 2D). However, in the histogram analysis (Figure 2E), the *Leptospira*-treated samples resulted in a sharper histogram profile with gray-scale mode of 72 and 66 for the 2,000 and 5,000 times magnifications, respectively, while the PBS samples resulted in a more diffuse histogram profile with mode = 85 for both the 2,000 and 5,000 times magnifications. Altogether, this indicates that there was a reduction in the number of adhered and spread platelets with a consequent increase in the area of the exposed coverslip in the samples treated with leptospires, suggesting adhered platelet detachment.

**FIGURE 3:**
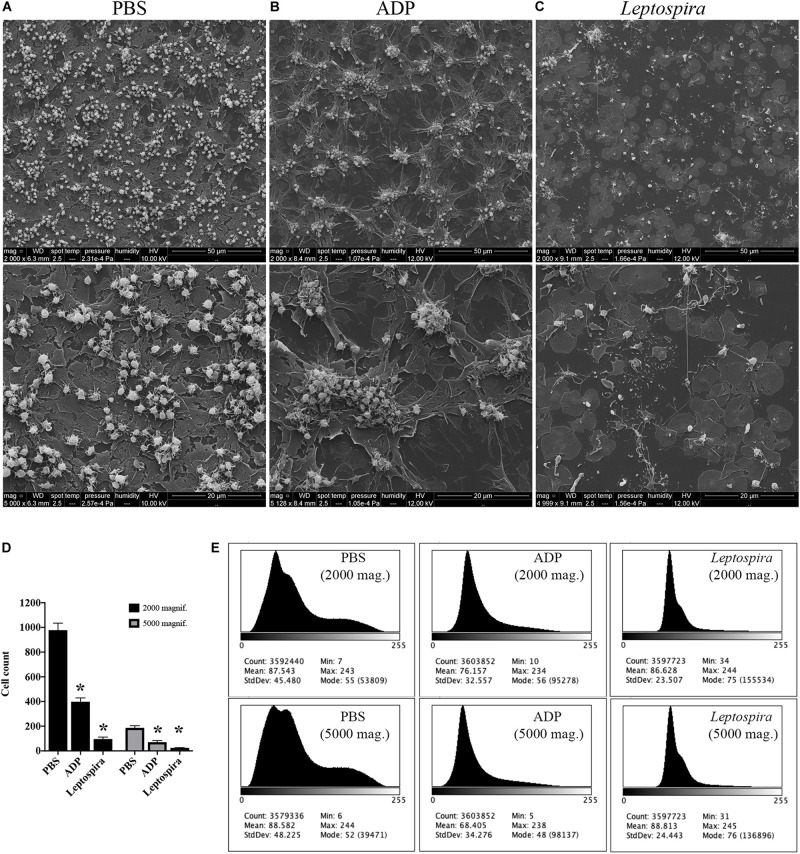
SEM showing the effect of leptospires on pre-adhered washed platelets. Washed platelets were allowed to adhere to coverslip glass surfaces, washed, and treated with PBS (**A**; negative control), ADP (**B**; positive control), or leptospires **(C)** for 30 min. After washing and fixation, samples were prepared for scanning electron microscopy. Upper panel: 2,000 magnification. Lower panel: 5,000 magnification. The figure shows representative panels of the samples. Two independent experiments were performed, with similar results. **(D)** Cell count of resting or dendritic platelets observed as individual cells in SEM images. The bars represent the mean ± standard deviation of two independent experiments. One-way ANOVA was used to compare the cell counts of ADP and *Leptospira*-treated samples with PBS-treated samples (*P > 0.0001). **(E)** Gray-scale histograms of the representative images, as analyzed by the software ImageJ.

**FIGURE 4:**
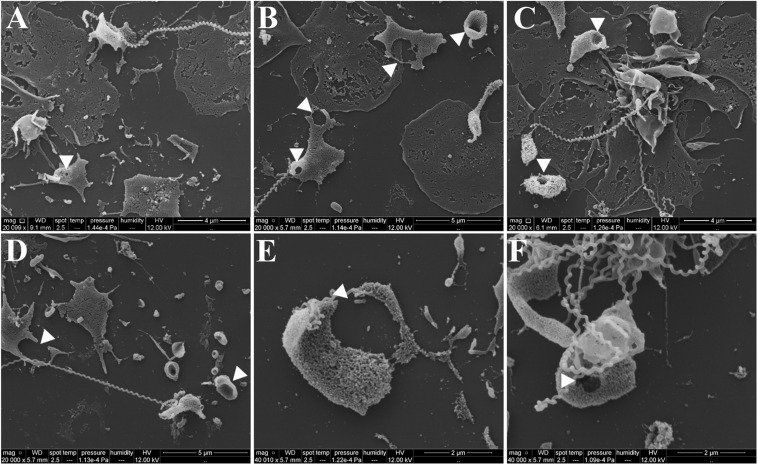
SEM showing platelet fragmentation and disruption after treatment with virulent leptospires. Pre-adhered washed platelets were incubated with virulent *L. interrogans*, resulting in platelet detachment, membrane disruption and fragmentation. Bacteria are seen interacting directly with the cells. The white arrowheads point to platelet perforations occurring at different levels. **(A–D)** 20,000 magnification. **(E,F)** 50,000 magnification. The figure shows representative panels of the samples. Two independent experiments were performed, with similar results.

To exclude the possible interference of plasma factors, the same experiment was performed with washed platelets (Figures 3, 4). The ADP-treated platelets resulted in the formation of aggregates (Figure 3B) and the negative control platelets presented with totally spread and dendritic-like morphology, covering all the glass surface (Figure 3A), with a consequent diffuse gray-scale histogram (Figure 3E). As we had to increase the number of cells to be able to see a significant number of platelets in the bacteria-treated sample, a second layer of dendritic platelets is observable above the layer of spread platelets in the negative control (Figure 3A). The samples incubated with leptospires showed a marked reduction in the number of cells, platelet detachment and disruption, visible by cell fragments on the coverslip surface (Figures 3C, 4). The cell count indicating significant less dendritic-platelet number in *Leptospira*-treated samples than in negative-control (PBS) or positive-control (ADP) samples (Figure 3D), as well as the sharper gray-scale histogram constrained to the darker hue (clear background without spread platelets) in the samples incubated with bacteria (Figure 3E), corroborate the observational interpretation of the images. The formation of platelet-bacteria clusters was less prominent when compared to the experiment with platelets in PRP, while the platelet disorganization and fragmentation was more pronounced (Figure 3C). Bacteria clearly interacting with platelets are visible in higher magnification, and the striking outcome of platelet fragmentation and perforation (white arrowheads, Figure 4) was consistently observed after incubation with virulent leptospires.

**FIGURE 5:**
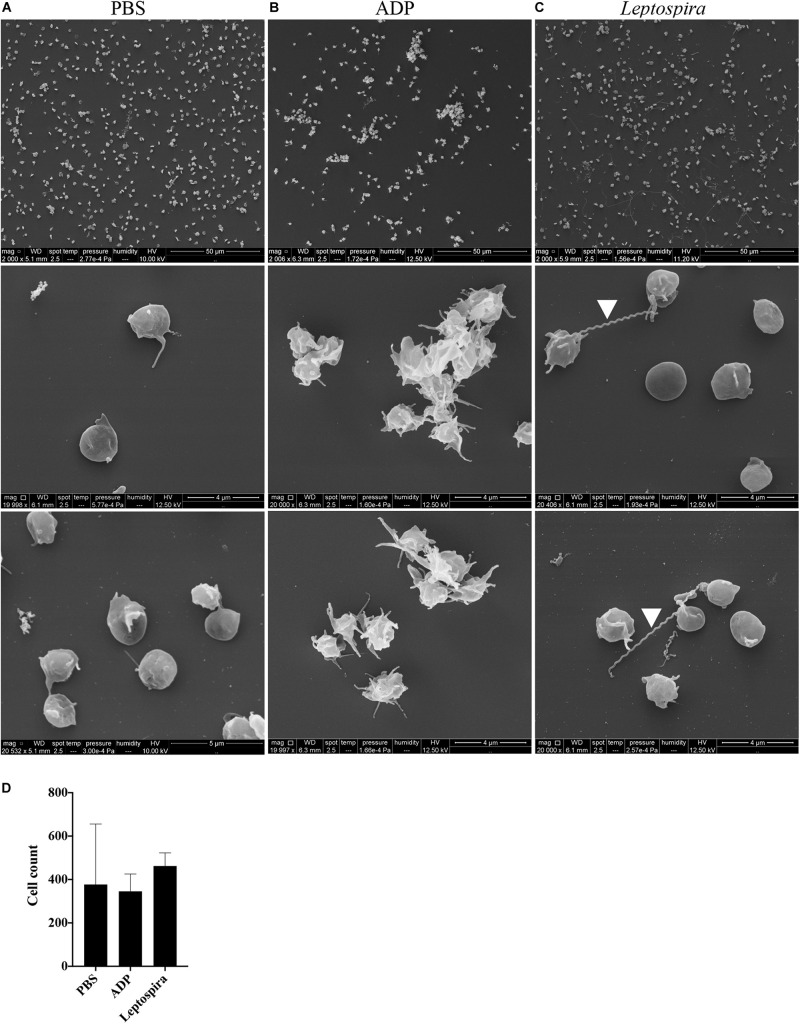
SEM of platelets incubated in suspension with different stimuli. Washed platelets were incubated in suspension with buffer alone (**A**, negative control), ADP (**B**, positive control) or live virulent *L. interrogans* serovar Copenhageni cells **(C)** for 30 min. After fixation onto glass coverslips, the samples were prepared for SEM and visualized at 2,000 (upper panel) and 20,000 magnification (middle and lower panels). The figure shows representative figures of the samples. Two independent experiments were performed, with similar results. **(D)** Platelet count in the 2,000 magnification panels. The bars represent the mean ± standard deviation of two independent experiments.

Alternatively, the interaction of washed platelets and leptospires was performed in suspension. When treated with buffer, platelets presented preferentially isolated, in the discoid form and with a smooth surface, compatible with a non-activated morphology (Figure 5A). Platelets activated with the ADP agonist formed aggregates, comprised by dendritic-like cells with a smooth surface, appropriate of an activated platelet morphology (Figure 5B). When the washed platelets (1 × 10^8^/mL) were treated with leptospires (1 × 10^7^/mL), platelets were predominantly isolated with morphology compatible with minimal activation (Figure 5C). Direct platelet-bacteria interactions are visible (leptospires are indicated by white arrowheads). Cell count analysis shows that platelet numbers are similar between the different treatments (Figure 5D).

**FIGURE 6:**
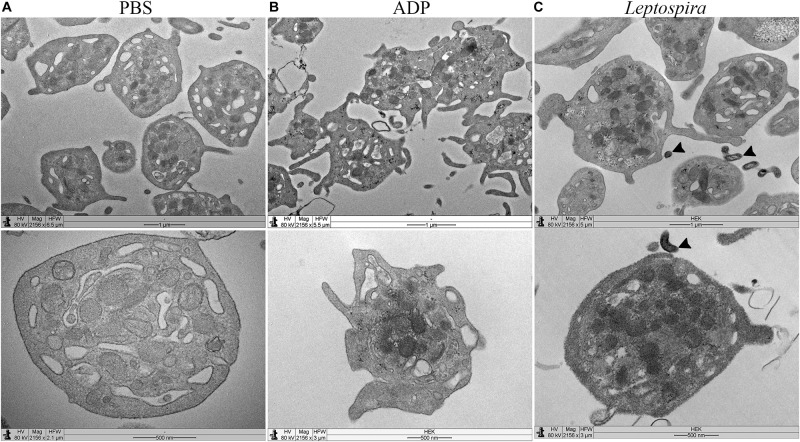
TEM of platelets incubated in suspension with different stimuli. Washed platelets were incubated in suspension with buffer alone (**A**, negative control for platelet activation), ADP (**B**, positive control for platelet activation), or live virulent *L. interrogans* serovar Copenhageni cells **(C)** for 30 min. After fixation, the samples were prepared for TEM and visualized with a LEO 906 E (Zeiss) transmission electron microscope.

Platelet-leptospiral interactions in suspension were further analyzed by TEM. When treated with PBS, the platelets were isolated, in discoid form, with a preserved membrane and granules and organelles distributed throughout the cell (Figure 6A). Platelets treated with ADP (Figure 6B) presented dendritic-like morphology with filopodia projections, preserved membrane and organelles and granules concentrated in the center of the cell body; different levels of degranulation and platelet-platelet aggregation were observed. The platelets (1 × 10^8^/mL) treated with leptospires (1 × 10^7^/mL) were isolated, and with morphology compatible with low level of activation and degranulation (Figure 6C). Platelet microvesiculation and disruption were consistently found. Direct interactions between *Leptospira* and platelets were observed (Figure 6C – black arrowheads).

### Platelets Remain Functional After Incubation With Leptospires

To evaluate whether platelets remain functional after *in vitro* treatment with leptospires, PRP was incubated with the bacteria in suspension for 30 min and then post-stimulated with ADP. The platelet reactivity, measured by the maximum percentage of aggregation, was not statistically different between platelets incubated with *L. interrogans* (80.50 ± 5.80) and control samples (platelets incubated with PBS only) (89.50 ± 4.79). The results suggest that platelets retain the ability to form aggregates after incubation with the bacteria in the assayed *in vitro* conditions.

**FIGURE 7:**
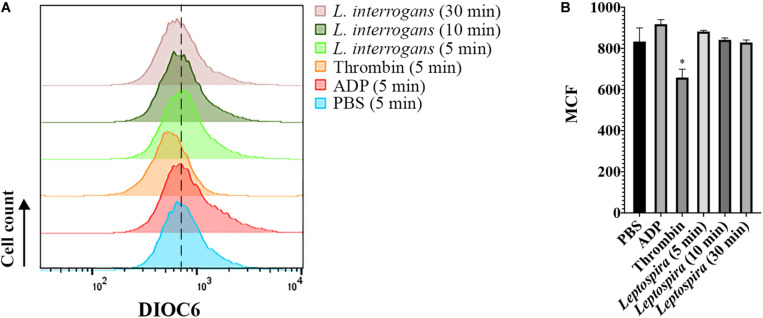
Flow cytometry analysis of platelet mitochondrial transmembrane potential after different stimuli. Platelets in PRP were incubated with the addition of PBS (negative control), ADP, thrombin, or virulent leptospires (2 × 10^6^/mL) for 5, 10, or 30 min, with the addition of the fluorescent dye DIOC6(3). Platelets were marked with CD41a-APC and the results represent only the platelet gate. **(A)** histogram of the DIOC6(3) detection on the CD41a-positive gate. The dashed line was included to aid in the interpretation of the histogram displacement. Data are representative of four independent experiments, with four different healthy blood donors. **(B)** Mean Channel Intensity (MCF) of each different sample. The bars represent the mean ± standard deviation of two independent experiments. For each sample, 20,000 events were collected. One-way ANOVA analysis was performed to compare the different samples with the negative control (PBS). *P > 0.0002.

Additionally, we evaluated whether leptospires trigger platelet apoptosis by flow cytometry. It is known that treatment of human platelets with strong agonists, such as thrombin, induces platelet apoptosis events, including mitochondrial transmembrane potential (Δψm) depolarization (Leytin et al., 2006). The platelet mitochondrial accumulation of the fluorescent dye DIOC6(3) was used as an indicator of the Δψm. While the platelets treated with the strong activator thrombin resulted in the depolarization of the Δψm, indicated by the reduced DIOC6(3) MCF, platelets treated with leptospires showed values similar to the PBS-treated negative control (Figure 7). Therefore, our results further suggest that platelets remain functional after incubation with *L. interrogans in vitro* in the assayed conditions.

## Discussion

Platelet adhesion and activation are independent processes mediated by different bacterial components and different platelet receptors. The bacterial ability to generate an intracellular signaling response after binding to platelets determines the consequence of the interaction. Bacteria can have an activating effect, an adhesive effect, both or neither. Bacteria can also induce platelet clearance, lysis, or apoptosis via different mechanisms. Novel clinical insights support that platelet activation or inhibition can be either harmful or protective, depending on the pathophysiological context (Nicolai et al., 2019).

The importance of direct bacteria–platelet interactions in disease pathogenesis is yet not fully understood. In recent years, studies have explored the mechanisms different species use to adhere to and activate platelets (Guo and Rondina, 2019). For example, contact of bacteria with platelets appears to be critical in the pathogenesis of sepsis since thrombocytopenia is directly related to disease severity and mortality. Sepsis thrombocytopenia usually occurs by increased platelet activation by the pathogen, cytotoxic effects, and induction of platelet apoptosis (Speth et al., 2013; Naime et al., 2018). These studies are providing insights into bacterial pathogenesis and allowing identification of targets for novel therapeutics against vascular infection.

Thrombocytopenia is a frequent finding in severe leptospirosis patients, and is associated with occurrence of hemorrhages and worse prognosis (Nicodemo et al., 1990; Wagenaar et al., 2007; Daher et al., 2014). The hypotheses to explain the mechanisms of platelet depletion in leptospirosis include (i) disseminated intravascular activation (DIC), (ii) platelet death induced by leptospiral toxins, (iii) decreased production of platelets by the bone marrow megakaryocytes, (iv) platelet over-activation by bacterial components leading to platelet consumption, and (vi) platelet clearance mediated by platelet autoantibodies. However, it remains unknown whether pathogenic *L. interrogans* can activate, inhibit, or kill platelets, and the respective mechanisms (Vieira et al., 2020). The present study presents evidence suggesting that virulent leptospires do not activate and induce cytotoxic effects in human platelets *in vitro*.

Our results indicate that leptospires alone cannot induce human platelets to form aggregates *in vitro*, what is in accordance with previous studies (Isogai et al., 1997). The aggregation results are further corroborated by the absence of the integrin GPIIbIIIa (CD41/61) activation in platelets incubated with leptospires. Upon platelet activation by ADP, thrombin, or other agonists, inside-out signals lead to GPIIbIIIa conformational changes from a resting conformation to an activated extended conformation, exposing a binding site which interacts with fibrinogen. Consequently, GPIIbIIIa forms a molecular bridge between adjacent activated platelets to form aggregates, by binding to fibrinogen. Changes in the state of GPIIbIIIa activation closely correlate with the degree of platelet-platelet aggregation (Frelinger, 2018).

Studies of platelet function and thrombocytopenia during leptospirosis in animal models of infection are limited, but indicate that platelet aggregation in circulation is not a major cause of platelet depletion. Evidence from the guinea pig model of experimental infection suggest that thrombocytopenia is caused by increased platelet clearance rather than aggregation in the circulation (Yang et al., 2006). Our results showing that platelets retain the ability to form aggregates after incubation with the bacteria in the assayed *in vitro* conditions, corroborates these *in vivo* observations.

Our results indicate that platelets do not secrete the content of intracellular granules after stimulation with live leptospires *in vitro*, as no changes in the light scattering characteristics by flow cytometry were observed, neither exposure of granule-derived molecules on the platelet membrane. Upon platelet activation, fusion of alpha-granules with the membrane results in P-selectin exposure on the platelet surface. P-selectin is therefore frequently used as a marker of alpha-granule secretion. The primary role of the exposed P-selectin is to mediate the platelet interactions with leukocytes, recruiting these cells to the site of vascular injury. Hence, our results suggest that leptospires might not directly activate the platelet response *in vitro*, as no P-selectin was detected on the bacteria-stimulated platelets. CD63, a lysosome and dense granule marker, was also not found on the surface of platelets after treatment with *L. interrogans*.

Altogether, our SEM analysis corroborate the lack of platelet activation and aggregation by leptospires, and further suggest that bacteria induce pre-adhered platelet detachment, disruption and fragmentation. The analysis of the gray-scale histograms was used to indirectly quantify the adhered platelets, and therefore relates to the platelet detachment. The individual platelets on the over layer or forming aggregates were also counted. Both in pre-adhered PRP and washed platelets samples treated with leptospires, there was a reduction in the total number of platelets: in PRP, mainly the adhered platelets were lost, while in washed platelets both adhered and over-layered platelets were lost. In the washed platelets treated with leptospires, increased platelet fragmentation was also observed in comparison to the PRP sample. In the presence of soluble factors in the plasma (PRP), the leptospires and platelets appear to form clusters or microaggregates. We speculate that soluble plasma factors might protect platelets from detachment and that the *Leptospira*-platelet microaggregates might be the result of fibrin generation, as these bacteria activate the coagulation cascade by various platelet-independent mechanisms *in vitro* (Vieira et al., 2016, 2017, 2020). Microaggregation of platelets treated with leptospires was not observed in the flow cytometry studies because platelets were diluted and not under stirring conditions to avoid contact (and aggregation). Similarly, ADP treatment resulted only in shape-change and not aggregation.

The TEM experiments further support the minimal platelet activation and degranulation induced by leptospires, and direct bacteria-platelet interactions. The morphology of platelets treated with leptospires is similar to the observed in the PSB samples, while ADP-treated platelets show marked degranulation and cellular extensions, as expected.

Furthermore, it is known that strong activation of platelets with agonists such as thrombin triggers platelet apoptotic events, with the exposure of activation markers (P-selectin, phosphatidylserine, etc.), activation of caspases and depolarization of Δψm. Our results indicating that platelets treated with leptospires do not undergo Δψm depolarization, further corroborates the lack of platelet activation and a different outcome than apoptosis for those cells.

Platelet dysfunction as a consequence of interaction with leptospires has been previously reported. The recombinant proteins BatA and BatB, which contain von Willebrand factor type A domain and MIDAS motif, were shown to bind to GPIb platelet receptor, inhibiting platelet aggregation mediated by its ligands, thrombin and von Willebrand factor (Fang et al., 2018; Passalia et al., 2020). Tunjungputri et al. (2017) observed reduced reactivity to *ex vivo* activation of platelets from patients with probable leptospirosis (Tunjungputri et al., 2017). The same platelet effect is observed in patients with other hemorrhagic infectious diseases, such as dengue (Michels et al., 2014).

Our data strongly suggest that virulent leptospires stimulate human platelet disruption. These effects seem to be independent of platelet degranulation and activation. The individual bacterial components responsible for the observed effects in platelets are currently being investigated, along with the molecular mechanisms of bacteria-platelet interactions and signaling. However, we acknowledge that the *in vitro* analysis is a limitation of our study. Previous studies have demonstrated that platelets form aggregates (i.e., are activated) by neutrophils primed with leptospiral lipopolysaccharide (Isogai et al., 1997), denoting that a more complex interplay might occur *in vivo*. Our results confirm the direct interactions of leptospires and platelets, and strongly suggest that these interactions might result in platelet dysfunction and thrombocytopenia observed during leptospirosis. The cytotoxic effect of leptospires in platelets might significantly contribute to the leptospirosis thrombocytopenia, imposing significant pathophysiological consequences in the illness.

## Data Availability Statement

All datasets presented in this study are included in the article/supplementary material.

## Ethics Statement

The studies involving human participants were reviewed and approved by the Ethics Committee on Research of Instituto de Assistência Médica ao Servidor Público Estadual (IAMSPE), São Paulo, Brazil – protocol 2973410. The patients/participants provided their written informed consent to participate in this study.

## Author Contributions

MV conceived, designed, and performed the experiments, analyzed the data, and wrote the manuscript. MV and AN contributed with reagents, materials, and analysis tools and revised the manuscript. Both authors contributed to the article and approved the submitted version.

## Conflict of Interest

The authors declare that the research was conducted in the absence of any commercial or financial relationships that could be construed as a potential conflict of interest.
